# A 6-year safety surveillance of 10-valent pneumococcal non-typeable *Haemophilus influenzae* protein D conjugate vaccine (PHiD-CV) in South Korea

**DOI:** 10.1080/21645515.2018.1502525

**Published:** 2018-08-29

**Authors:** Soon Min Lee, Jang Hoon Lee, Eun Song Song, Sung Jin Kim, Joon Hyung Kim, Rupert W. Jakes, Raghavendra Devadiga, Moon Sung Park

**Affiliations:** a Department of Pediatrics, Gangnam Severance Hospital, Yonsei University College of Medicine, Seoul, Korea; b Department of Pediatrics, Ajou University School of Medicine, Suwon, Korea; c Department of Pediatrics, Chonnam National University Medical School, Gwangju, Korea; d GSK, Seoul, Korea; e GSK, Singapore, Singapore; f GSK, Bangalore, India

**Keywords:** 10-valent pneumococcal conjugate vaccine, PHiD-CV, safety, Korea

## Abstract

In 2010, Korea introduced 10-valent pneumococcal conjugate vaccine for children aged 6 weeks to 5 years against invasive disease caused by *Streptococcus pneumoniae* serotypes 1, 4, 5, 6B, 7F, 9V, 14, 18C, 19F, 23F and cross-reactive 19A. The aim of this 6-year real-world study of 646 healthy Korean children from 16 centers vaccinated in routine practice is to monitor vaccine safety, as per Ministry of Food and Drug Safety regulations. Around 50% had a past or existing medical condition, 19.3% an existing condition and 7.6% received concomitant medication). Total of 489 recorded adverse events (AEs) were reported in 274 infants; 86% were mild and the rest moderate, only three were reported as serious. Most AEs (97.8%) were not related to vaccination; one case of injection-site swelling and of fever was related, two cases of fever were probably related, five cases of fever and one case each of diarrhea and coughing were possibly related. None of the serious AEs were related to vaccination. Of 11 adverse drug reactions (ADRs) in 10 subjects, none were serious. Overall, 263 subjects (40.7%) received medication (mainly antibiotics or antipyretics) for the treatment of an AE, of which 6 subjects were treated for an ADR. There was no difference in the incidence of AEs according to age, sex or concomitant vaccination. Subjects with an existing medical condition had significantly more AEs than those without any conditions (p = 0.03), but no differences regarding ADRs. Four-dose vaccination with PHiD-CV appears to have a clinically-acceptable safety profile for Korean children.

**ClinicalTrials.gov identifier**: NCT01248988

## Introduction

Pneumococcal disease, caused by *Streptococcus pneumoniae* (*S. pneumoniae*) spreads by droplets of respiratory secretions^1^ and can cause different types of illnesses, including meningitis, sepsis, pneumonia, and otitis media.^2^ Severe pneumococcal disease accounts for around 14.5 million cases globally each year among children under five years old,^3^ resulting in almost 500,000 deaths, mostly in developing countries.^4^ Routine pneumococcal conjugate vaccination programs for infants have dramatically decreased invasive pneumococcal disease incidence, with near elimination of disease caused by vaccine serotypes in some places.^5^

The pneumococcus comprises over 90 serotypes,^4^ each producing a unique polysaccharide, called the capsule, which is a major virulence factor and the target of pneumococcal conjugate vaccines.^6^ Two pneumococcal conjugate vaccines have market authorization; *Prevenar* (also *Prevnar*, PCV13, Pfizer) is a 13-valent vaccine conjugated to CRM197 protein, and *Synflorix* (GSK) is a 10-valent vaccine using protein D, derived from non-typeable *Haemophilus influenzae*, as a carrier protein for 8 out of the 10 serotypes.

The 10-valent pneumococcal non-typeable *Haemophilus influenzae* protein D conjugate vaccine (PHiD-CV, *Synflorix*, GSK) containing pneumococcal serotypes 1, 4, 5, 6B, 7F, 9V, 14, 18C, 19F and 23F was registered in Korea in March 2010.^7^ The vaccine is indicated for active immunization of infants and children from 6 weeks up to 5 years of age against invasive disease, pneumonia and acute otitis media caused by *S. pneumoniae* serotypes 1, 4, 5, 6B, 7F, 9V, 14, 18C, 19F, and 23F.^8^ In May 2016, the European Medicines Agency updated the label to include effectiveness for cross-reactive serotype 19A invasive pneumococcal disease (IPD), based on three postmarketing observational studies conducted in Brazil, Finland, and Canada (Quebec).^8,9^ A matched case-control study in Brazil^10^, reported a significant decrease in IPD due to any vaccine serotype, and 19A serotype (i.e., adjusted effectiveness 82.2% (95% confidence interval [CI] 10.7, 96.4)).^10,11^ In Finland^12^, a comparison of before and after introducing the vaccine into the national immunization program found a significant decrease of 80% (95% CI 72, 85) in the incidence of any culture-confirmed IPD, including IPD due to serotype 19A.^11,12^ In Quebec^13^, a decrease of incidence in vaccine serotype IPD as well as in serotype 19A IPD was observed, showing 71% (95% CI 24, 89) vaccine effectiveness.^11,13^

Following registration, Korea’s Ministry of Food and Drug Safety (MFDS) requires postmarketing surveillance (PMS) to collect safety information in at least 600 Korean infants and children over a 6-year period.^14,15^ Therefore the aim of this 6-year PMS study was to monitor the safety of PHiD-CV vaccination given according to Korean Prescribing Information (PI) in routine practice.^16^

## Results

### Participants

Subjects were recruited from 16 centers in Korea including university hospitals, general hospitals, women’s hospitals and paediatric hospitals/clinics. A total of 647 subjects were enrolled, of which 646 subjects were included in the safety analysis. One subject was excluded as the age of this subject was not within the protocol-defined range (i.e. primary vaccination at 2, 4 and 6 months of age, six weeks to five years old at first dose).

The majority of subjects enrolled in the study when receiving their first dose (519 subjects, 80.3%), 43 subjects (6.7%) enrolled when receiving their second dose, 54 subjects (8.4%) enrolled when receiving their third dose and 30 subjects (4.6%) enrolled when receiving their booster dose. As a result, among the 646 subjects enrolled; 519 subjects received dose one, 466 subjects received dose two (includes subjects who enrolled when receiving doses one and two), 399 subjects received dose three (includes subjects who enrolled when receiving doses one, two or three) and 57 subjects received a booster dose (includes subjects who enrolled when receiving doses one, two, three or booster).

### Descriptive data

All subjects were of Korean heritage, 52.2% were male. The mean age of subjects at dose 1 was 11.7 weeks (Standard deviation [SD] 9.7 weeks), at dose 2 was 20.7 weeks (SD 7.9 weeks), at dose 3 was 29.7 weeks (SD 5.4 weeks) and for the booster dose was 61.4 weeks (SD 6.6 weeks).

Overall, 302 subjects (46.7%) had a past medical condition; mainly respiratory, thoracic and mediastinal disorders (20.7%, e.g., upper respiratory infection), skin and subcutaneous tissue (12.2%, e.g. atopic or seborrheic dermatitis), hepatobiliary (10.1%, e.g., neonatal jaundice) or gastrointestinal disorders (7.1%, e.g., acute gastroenteritis), and 21 were premature babies. Of the 646 subjects, 125 (19.3%) had an existing medical condition; mainly respiratory, thoracic and mediastinal disorders (6.7%, e.g., asthma or upper respiratory infection) or skin and subcutaneous tissue disorders (4.6%, e.g., atopic dermatitis or miliaria).

During the entire study period, 312 subjects (48.3%) received concomitant medication; out of these, 49 were not treated for AE. Overall, 134 (20.7%) subjects received antibiotics and 97 (15.0%) received antipyretics. The majority of subjects on concomitant medication reported using respiratory medication (246, 38.1%), alimentary tract and metabolism medication (183, 28.3%) and anti-infectives (140, 21.7%).

The percentage of subjects receiving concomitant medication by visit was; 28.5% following dose one, 29.4% following dose two, 30.6% following dose three and 36.8% following the booster dose visit. The majority of subjects (630 subjects [97.5%]) received concomitant vaccinations most frequently including; Haemophilus influenzae b vaccine (609 subjects), rotavirus vaccine (434 subjects), hepatitis B vaccine (189 subjects) and diphtheria tetanus and acellular pertussis vaccine (179 subjects).

### Safety data

A total of 489 adverse events (AEs) were reported by 274 subjects (42.4%) and 11 adverse drug reactions (ADRs) were reported by 10 subjects (1.6%) during the study period. Three subjects (0.5%) reported three serious adverse events (SAEs) (i.e., erythema multiforme, urinary tract infection and pneumonia), which were assessed by investigators as unlikely to be related to PHiD-CV vaccination. There were no serious ADRs reported (Table 1).

**Table 1.** Summary of events over the study period (Total vaccinated cohort = 646).

AE characteristics	n* (AEs)	n (subjects)	% (subjects)
AE total	**489**	**274**	**42.41**
Unexpected AE	422	251	38.85
Mild	419	242^£^	37.46^£^
Moderate	67	50^£^	7.74^£^
Severe	0	0	0.00
SAE total	**3**	**3**	**0.46**
Unexpected SAE	3	3	0.46
ADR total	**11**	**10**	**1.55**
Unexpected ADR	1	1	0.15
SADR total	**0**	**0**	**0.00**
Unexpected SADR	0	0	0.00

^£^Same subjects can be included under both Mild and Moderate. Severity is not captured for 3 AEs whose details are missing.

ADR: adverse drug reaction; AE: adverse event; n*: number of events; n: number of subjects with at least one symptom; SADR: serious adverse drug reaction; SAE: serious adverse event

When assessing the occurrence of events after each dose, post-dose one; one subject reported an SAE, 114 subjects (22.0%) reported AEs, and seven subjects reported eight ADRs. Post-dose two; two subjects reported two SAEs, 119 subjects (25.5%) reported AEs, and, two subjects reported two ADRs. Post-dose three; 99 subjects (24.8%) reported AEs, and one subject had an ADR. Following the booster dose; 16 subjects (28.1%) reported AEs.

Most AEs (422) and one ADR (i.e., coughing) were considered unexpected (i.e. not in the PI). The most frequently reported unexpected AEs were respiratory disorders in 34.5% of subjects (95% Confidence Interval [CI] 30.9, 38.3); 327 cases in 223 subjects, and gastrointestinal disorders in 4.8% of subjects (95% CI 3.3, 6.7); 32 cases in 21 subjects. Only 1 unexpected case (i.e., coughing) was considered possibly related to vaccination. See Table 2 for details of the most frequent unexpected AEs reported in over 10 cases.

**Table 2.** Most frequent unexpected AEs reported in ≥ 10 cases (Total vaccinated cohort = 646).

Unexpected AEs	n* (AEs)	n (subjects)	% (subjects)	Related to vaccination
Respiratory disorders				
Bronchiolitis	40	34	5.3%	Unlikely
Bronchitis	44	37	5.7%	Unlikely
Coughing	11	11	1.7%	1 Possibly 10 Unlikely
Nasopharyngitis	54	43	6.7%	Unlikely
Respiratory disorder	27	16	2.5%	Unlikely
Rhinitis	10	10	1.5%	Unlikely
Rhinorrhea	12	11	1.7%	Unlikely
Upper respiratory tract infection	96	81	12.5%	Unlikely
Gastrointestinal disorders				
Gastroenteritis	16	16	2.5%	Unlikely
Resistance mechanism disorders				
Otitis media	21	17	2.6%	Unlikely
Vision disorders				
Conjunctivitis	10	10	1.5%	Unlikely

AE: adverse event; n*: number of events; n: number of subjects with at least one symptom; Possibly: temporal sequence of the administration and use of PHiD-CV appropriate but it could also be explained by other medications, chemical substances or concomitant disease; Unlikely: transitory case which may not have had causality with the administration and use of PHiD-CV, also explained reasonably by other medications, chemical substances or latent disease.

Among the three subjects with an SAE; pneumonia lasting 29 days, and erythema multiforme lasting nine days, were reported five and 11 days respectively after receiving dose two of PHiD-CV. Urinary tract infection, lasting four days, was reported seven days after dose one of PHiD-CV. All of the SAEs were assessed by the investigators as unlikely to be related to PHiD-CV vaccination, and, all resolved by the end of the study.

Among the 489 AEs, 419 (85.7%) were graded as mild in intensity and 67 (13.7%) were graded as moderate in intensity by investigators. There were no severe-intensity AEs (Table 1). The most frequent moderate AEs (≥ 5 cases) were; 11/96 cases (11.5%) of upper respiratory tract infection, 5/16 cases (31.3%) of gastroenteritis, 18/44 cases (40.9%) of bronchitis, and, 8/40 cases (20.0%) of bronchiolitis.

Investigators assessed whether AEs were caused by vaccination as follows, according to MFDS requirements. Most of the AEs (478, 97.8%) reported during the entire study period were assessed as *unlikely* to be related to vaccination. Of the 11 ADRs; two cases each (0.4%) were *certainly* and *probably* related to vaccination, and, seven cases (1.4%) were *possibly* related to vaccination. There were no AEs with causality classed as *conditional* or as *unassessable/unclassifiable*. Vaccination was certain to have caused a case of injection site swelling and of fever, probably caused two cases of fever, and possibly caused five cases of fever and one case each of diarrhea and coughing. See full details in Appendix Table 1.

### Influencing factors for AEs and ADRs

There was no significant difference in outcomes stratified by gender (p = 0.74 for AEs, p = 0.10 for ADRs); by age < 1 year old versus ≥ 1 year old (p = 0.65 for AEs, no ADRs in ≥ 1 year olds), for those with or without a previous medical condition (p = 0.35 for AEs, p = 0.09 for ADRs), or, by concomitant vaccination (p = 0.53). Subjects with a current medical condition had significantly more AEs than subjects without it (51.2% vs 40.3%, p = 0.03) although with no significant difference for ADRs). As most medication use in the study was related to AEs (i.e., 263/312 subjects with medication use was for AE/ADRs), those on medication were much more likely to have had an AE than those with no medication use (86.2% versus 1.5%, p < 0.0001) (Table 3).

**Table 3.** Analysis of factors influencing AEs and ADRs (Total vaccinated cohort = 646).

	n* (AEs)	n (subjects)	p value	n* (ADRs)	n (subjects)	p value
Gender						
Male N = 337	238	145	0.743	3	3	0.097
Female N = 309	251	129		8	7	
Age						
< 1 year old N = 605	465	258	0.650	11	10	-
≥ 1 year old N = 41	24	16		-	-	
Previous medical condition						
Yes N = 302	253	134	0.346	8	7	0.088
No N = 344	236	140		3	3	
Existing medical condition						
Yes N = 125	117	64	0.027	4	4	0.080
No N = 521	372	210		7	6	
Concomitant medication (for treatment of AEs or other)						
Yes N = 312	482	269	< 0.0001	8	7	0.101
No N = 334	7	5		3	3	
Medication used for AE treatment						
Yes N = 263	472	263	< 0.0001	7	6	0.118
No N = 383	17	11		4	4	
Concomitant antibiotics						
Yes N = 134	271	131	< 0.0001	2	2	0.304
No N = 512	218	143		9	8	
Concomitant antipyretics						
Yes N = 97	179	85	< 0.0001	3	3	0.130
No N = 549	310	189		8	7	
Concomitant vaccination						
Yes N = 630	475	266	0.534	11	10	-
No N = 16	14	8		-	-	

ADR: adverse drug reaction; AE: adverse event; N: number of vaccinated subjects; n*: number of events; n = number of subjects with at least one symptom

**Figure 1.**
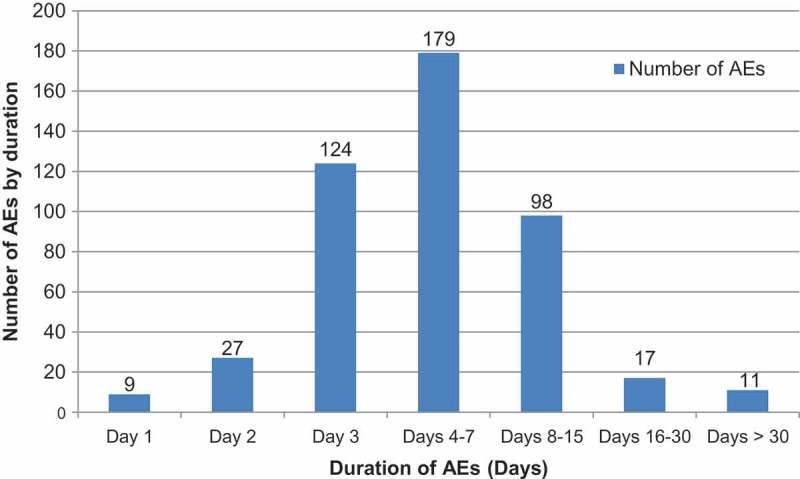
Duration of adverse events (days) (Total vaccinated cohort = 646). AE: adverse event

As for the duration of AEs, 38.5% (n = 179) lasted for 4–7 days; the most frequent types of AE in this category were 31.3% (n = 30/96) of upper respiratory tract infections, 44.4% (n = 24/54) of nasopharyngitis cases, 41.9% (n = 18/44) of bronchitis cases, 28.2% (n = 11/40) of bronchiolitis cases, 37% (n = 10/27) of respiratory disorders, and 50% (n = 11/22) of diarrhea cases (Figure 1). The AEs that lasted for over 30 days (n = 11, 2.4%) included; one case each of upper respiratory tract infection, rhinitis, respiratory disorder, pneumonia, nasopharyngitis, fracture, contact dermatitis, atopic dermatitis, and, three cases of bronchitis. All reported AEs had resolved by the end of the study, except for 4 cases (bronchitis, atopic dermatitis, otitis media and rhinorrhea) for which the outcome was unknown.

## Discussion

The results of this study showed that four-dose vaccination with PHiD-CV was well tolerated in Korean infants. A total of 646 subjects from 16 hospitals in Korea were included in the safety analysis over a 6-year period and have particular importance in that they covered different levels of medical and regional support. Data from around two-thirds (≥ 399) of subjects were analysed for each primary vaccine dose, and from 57 subjects for the booster dose. Almost all subjects received other vaccinations during the study period, as expected. The similar safety and reactogenicity profiles of PHiD-CV were confirmed when administered for primary and booster vaccination of children from 6 countries, using a range of vaccination schedules and coadministered with other routinely used pediatric vaccines. As this was a real-world study, around 19% of subjects had an existing condition, around 8% were receiving other medication and 21 premature babies were included in the study.

A total of 489 AEs were recorded in 274 subjects; 97.5% were not considered to be related to vaccination. Most of them were infectious events including respiratory, gastrointestinal and other systems’ disorders considered as existing infections or conditions related to an underlying disease by the study investigators. Most AEs (85.7%) were graded as mild, and the rest as moderate in intensity. Vaccination with PHiD-CV caused one case of injection-site swelling and of fever, probably caused two cases of fever, and possibly caused five cases of fever and one each of diarrhea and coughing. There were three SAEs(i.e., erythema multiforme, urinary tract infection and pneumonia), but none were assessed as related to vaccination by the study investigators after the examination of the patients. There were 11 ADRs recorded in 10 subjects, none of which were serious ADRs. The incidence is relatively low compared to previous reports. In other studies, overall, SAEs were reported by 150 of 2,996 subjects (5%). Redness was the most commonly reported solicited local symptom. Pain or swelling at the injection site were the most commonly reported grade 3 local symptoms.^17^ As for the reports from the European Union Summary of Product Characteristics (EU SPC), following administration of PHiD-CV in the primary infant vaccination series, the most frequently reported AEs were irritability (55% of all doses), and redness at the injection site (41% of all doses). Irritability (53%) and pain at the injection site (51%) were the most common AEs after the booster dose.^8^ These AEs were transient and mild to moderate in severity in most cases. There was also no increase in the frequency or severity of these events with subsequent doses of PHiD-CV during primary vaccination.^17^ According to the recommendation from EU SPC, the use of prophylactic antipyretics is recommended when PHiD-CV is given concomitantly with vaccines containing whole cell pertussis, and in children with seizure disorders or with a prior history of febrile seizures.^8^ During this entire study period, 97 (15.0%) received antipyretics. No significant differences were found by gender, age, concomitant vaccinations, or for past medical conditions in terms of risk of developing an AE, however, significantly more subjects with an existing medical condition reported AEs compared with those without any condition. Care should be taken when administering PHiD-CV to infants with comorbidities.

The results of this study were as expected, with no safety signals detected for rare or serious ADRs, and comparable to the safety findings of clinical trials of PHiD-CV.^8,11^ Safety results were also comparable to those seen in a phase III randomized clinical trial of PHiD-CV co-administered with the diphtheria, tetanus, acellular pertussis, hepatitis B virus, inactivated poliovirus and *Haemophilus influenzae* type b vaccine (DTPa-HBV-IPV/HiB) in Vietnamese infants; i.e., pain at injection site, irritability and fever were the most common local and general AEs, respectively, seen in the PHiD-CV group, with no SAEs causally related to the vaccine.^18^

PHiD-CV was proven to be well tolerated and generally as immunogenic in preterm infants as in term infants when given as a 3-dose primary vaccination followed by a booster dose. Incidences of fever and other solicited general symptoms were generally similar. Irritability was the most frequently reported solicited general AE for preterm infants after primary vaccination and the booster dose while upper respiratory tract infections were some of the most frequently reported unsolicited AEs.^19^

There were some limitations to this study. As this was an observational public health surveillance study, there was no randomization, blinding or control group.

A relatively small number of hospitals were included. A small number of subjects enrolled in the study after already receiving their first, second or third primary vaccination, therefore safety data following those vaccinations were not available. The number of subjects who received a booster dose was relatively small compared to those vaccinated with the primary series. Data were self-reported, which could have led to reporting bias; however, the proportion reporting a reaction was consistent with data from previous years. Despite the short timeframe for self-reporting of AEs after each dose, it is possible that incomplete information about medication or AEs was provided by parents/guardians. Some of the subjects included had existing conditions (mostly respiratory, thoracic or mediastinal disorders) for which it was not possible to determine the severity of illness, and some subjects were receiving concomitant medication other than for AEs. It was not possible to state whether these factors may have influenced the vaccination outcomes. Results should therefore be interpreted with caution and more data are needed in the future to confirm the safety outcomes observed for this group in this study.

## Conclusions

In conclusion, based on six years of safety data collected from 16 centers, four-dose vaccination with PHiD-CV appears to have a clinically-acceptable safety profile for children in Korea.

## Patients and methods

### Study design

A 6-year prospective, non-comparative, observational PMS study was conducted in 16 centers in Korea to actively collect data on the frequency and severity of adverse events (AEs) and serious adverse events (SAEs), following vaccination with PHiD-CV given in routine practice and according to Korean Prescribing Information (NCT01248988).

### Setting and population

Subjects attending a participating center for vaccination were enrolled if their parents/guardians gave consent and if they met the following inclusion and exclusion criteria. Subjects were included if; they received ≥ 1 dose of PHiD-CV as part of routine practice at a hospital or private clinic, were aged six weeks to five years old at first dose, and, their parent/guardian was likely to comply (according to the investigator) with completion of the diary cards. Subjects were excluded if; at the time of study entry they had any contraindication indicated in the local Prescribing Information, they had received any investigational or non-registered pneumococcal vaccine thirty days prior to the study start, or they had received a pneumococcal vaccine other than PHiD-CV. Informed consent was obtained from the parent/guardian of the child.

Subject numbers were assigned sequentially from a range of numbers allocated to each center.

### Ethics

The study was conducted in accordance with local regulatory requirements (MFDS). Parents/guardians of subjects provided written informed consent for the collection and handling of personal and safety information before conducting any study-specific procedures.

### Vaccination

Routine infant vaccination consisted of a three-dose primary vaccination series given at two, four and six months of age, followed by a booster dose at least six months after the last dose, preferably between 12–15 months of age. The same primary series and booster dose are recommended for preterm infants (27–36 week gestation). For older infants (> 7 months of age) and children up to five years old who were not previously vaccinated, the schedule consisted of a two-dose primary vaccination series, two months apart, followed by a booster dose in infants aged under 12 months of age.^16^

### Outcomes and measurements

Demographic data such as age, gender and race were recorded as well as any pre-existing conditions or signs and symptoms present prior to the start of the study. A history-directed medical examination and a physical examination of the subject was conducted. Treatment of any abnormality observed during this examination was performed according to local medical practice or by referral to an appropriate health care provider. Information regarding all vaccines administered since birth was collected. At each subsequent study visit, investigators recorded any concomitant medication, given during a 30-day period following administration of each dose. Concomitant medication could include any medication administered prophylactically in anticipation of a reaction to vaccination as well as any medication intended to treat an AE.

The oral, axillary, tympanic or rectal body temperature of subjects was measured prior to vaccination. Visits were rescheduled for subjects with fever (i.e., oral, axillary or tympanic temperature ≥ 37.5°C (99.5°F), or rectal temperature ≥ 38.0°C (100.4°F) or any clinical condition not suitable for vaccination. After completing any prerequisite procedures prior to vaccination, one dose of PHiD-CV was administered according to the PI (i.e., given by intramuscular injection, at the preferred sites of the anterolateral aspect of the thigh in infants or the deltoid muscle of the upper arm in young children). Vaccinees were closely monitored for ≥ 30 minutes in case of anaphylaxis.

Following vaccination and for a period of 30 days after each dose, the occurrence of any AEs was recorded by the parent/guardian in a diary card to be returned to the investigator at the next visit or by phone or mail. Subjects were followed until resolution of the AE. Any signs or symptoms perceived as serious were reported immediately to investigators. Investigators verified and discussed the recorded safety information against source documents and with the parent/guardian. The investigators then differentiated between *expected* AEs (i.e., reported in the PI) and *unexpected* AEs, for the 30-day follow-up period after each dose, and, recorded SAEs using the latest version of Korea’s PMS SAE Reporting Form, from the first dose in the study up to 30 days after the last dose.

#### Definition and assessment of AEs, ADRs and SAEs

An AE was defined as any untoward medical occurrence, temporally associated with the use of a medicinal product, whether or not considered related to the medicinal product. Symptoms were coded using the World Health Organization Adverse Reactions Terminology (WHO-ART 092 version); AEs mentioned in the PI were classed as ‘expected’, while all others were classed as ‘unexpected’.^20^ AEs possibly due to vaccination (so-called adverse drug reactions, ADR) were any AE with causality graded as “certainly” or “probably/likely” or “possibly” related to vaccination, or AEs that were “conditional/unclassified” or “unassessible/unclassifiable”. ADRs were also classed as expected or unexpected according to the events mentioned in the PI. A SAE was defined as any untoward medical occurrence that; resulted in death, was life-threatening, required hospitalization or prolongation of existing hospitalization, or resulted in disability or incapacity. An SADR was an SAE for which causality was classed as “certainly” or “probably/likely” or “possibly” related to vaccination, or SAEs that were “conditional/unclassified” or “unassessible/unclassifiable”.

Investigators assessed the intensity and causality of AEs and SAEs using their clinical judgement. Regarding causality, the most frequent unexpected AEs were mostly graded as ‘Unlikely’ to be related to vaccination (e.g., transitory AE which may not have had causality with the administration and use of PHiD-CV, or which can also be reasonably explained by other medications, chemical substances or latent disease), and one AE was graded as ‘Possibly’ related to vaccination (i.e., temporal sequence of the administration and use of PHiD-CV is appropriate but the AE could also be explained by other medications, chemical substances or concomitant disease). The maximum intensity over the duration of each event was classified as mild, moderate or severe. Mild events were; easily tolerated by the subject, caused minimal discomfort and did not interfere with everyday activities. Mild fever was 37.5°C to 38.0°C. Moderate events were sufficiently discomforting to interfere with normal everyday activities. Moderate fever was > 38°C to 39.0°C. Severe events prevented normal, everyday activities. Severe fever was > 39.0°C. The relationship between vaccination and the occurrence of AEs or SAEs was investigated, considering plausible alternative causes, the natural history of underlying diseases, concomitant therapy and other risk factors. A distinction was made between non-serious and serious AEs; for SAEs, additional

examinations and tests were performed to determine all possible contributing factors such as medical history, other medication or procedures, lack of efficacy of the vaccine(s), if applicable, erroneous administration and any other causes. As per the requirements of Korea’s MFDS, causality was classified on a six-point scale; from 1) *Certain* to 2) *Probable/Likely* to 3) *Possible* to 4) *Unlikely* to 5) *Conditional/unclassified* to 6) *Unassessable/unclassifiable*.^20^

### Statistical methods

Descriptive demographic characteristics of the study cohort were tabulated. Mean ages in weeks of subjects receiving each primary vaccination dose and booster dose were computed.

The safety analyses were conducted on the total vaccinated cohort. The AEs/SAEs were analyzed by their *expected* or *unexpected* status. Numbers of subjects by types of AEs were tabulated. The number and the percentage of any AE occurring within 30 days of each dose were tabulated; for each dose, for overall doses and per subject. The same calculations were done for any AEs according to severity and for those assessed as causally related to vaccination. The number and percentage of subjects with concomitant medication (and of doses of concomitant medication) were tabulated, after each vaccine dose and overall. SAEs and withdrawals due to AEs were described in detail.

Factors such as age, gender, existing medical conditions, concomitant medications, concomitant vaccination, and duration of follow-up were considered as factors that could affect AE outcomes. Results were stratified, using the Chi-squared test for significant differences (p values) for AEs and the Fisher’s Exact test for ADRs.

## Trade mark section

Synflorix is a trade mark owned by or licensed to the GSK group of companies. Prevnar 13 is a trade mark of Wyeth LLC.
